# Severe Hyponatremia in a Septic Patient With Bronchopneumonia: A Case Report

**DOI:** 10.7759/cureus.105640

**Published:** 2026-03-22

**Authors:** Nidhi Kakkar

**Affiliations:** 1 Department of Medicine for Older People, Stockport NHS Foundation Trust, Stockport, GBR

**Keywords:** aztreonam, hyponatremia, meropenem therapy, penicillin allergy, sepsis treatment

## Abstract

Hyponatremia is a common electrolyte abnormality encountered in hospitalized patients and may arise from a variety of etiologies, including infection, medications, endocrine disorders, and inappropriate fluid management. Severe hyponatremia can lead to significant neurological morbidity and requires careful evaluation and management. We report the case of a 72-year-old woman with a background of chronic obstructive pulmonary disease (COPD) and type 2 respiratory failure who developed persistent severe hyponatremia during treatment for sepsis secondary to bronchopneumonia. Despite treatment with broad-spectrum antibiotics and fluid restriction, the patient’s sodium remained low at approximately 116 mmol/L. Extensive investigations, including serum osmolality and serum cortisol, were within normal limits. Following the escalation of antibiotic therapy from aztreonam to meropenem and the removal of the fluid restriction, the patient’s sodium levels gradually improved over several days. This case highlights the importance of reassessing fluid management and ongoing sepsis as contributing factors in persistent hyponatremia.

## Introduction

Hyponatremia, defined as a serum sodium concentration below 135 mmol/L, is one of the most frequently encountered electrolyte abnormalities in hospitalized patients. Severe hyponatremia, particularly when sodium levels fall below 120 mmol/L, may lead to neurological complications, including confusion, seizures, and coma [1]. The etiology is often multifactorial and may include syndrome of inappropriate antidiuretic hormone secretion (SIADH), infections, medications, endocrine abnormalities, and disturbances in fluid balance [2].

Pulmonary infections, particularly pneumonia, are well-recognized precipitants of hyponatremia and may induce SIADH or other mechanisms that impair free water excretion. The evaluation of severe hyponatremia requires a systematic approach, including the assessment of serum osmolality, endocrine function, and fluid status [3].

In this report, we present a case of persistent severe hyponatremia in an elderly patient with sepsis secondary to bronchopneumonia, highlighting the challenges in diagnosis and management.

## Case presentation

A 72-year-old woman presented with features of sepsis secondary to bronchopneumonia. Her past medical history was significant for chronic obstructive pulmonary disease (COPD) and type 2 respiratory failure. She also had a documented allergy to penicillin.

On admission, she was commenced on intravenous teicoplanin due to concerns regarding bacterial infection and her penicillin allergy. As her inflammatory markers remained markedly elevated, antibiotic therapy was subsequently escalated to aztreonam.

Despite ongoing treatment, the patient’s C-reactive protein (CRP) remained significantly elevated, initially measuring approximately 301 mg/L and only decreasing to 196 mg/L. Concurrently, the patient was noted to have severe persistent hyponatremia, with serum sodium levels around 116 mmol/L.

Further investigations were undertaken to determine the underlying cause of the hyponatremia. Serum osmolality, serum cortisol, and other relevant biochemical investigations were performed and were found to be within normal limits [4]. The patient was initially managed with a fluid restriction of approximately 1.5 L per day due to concerns regarding possible SIADH.

Given the lack of significant improvement in inflammatory markers and persistent infection, aztreonam was discontinued, and antibiotic therapy was escalated to meropenem. At the same time, the previously instituted fluid restriction was removed [5].

Following these changes in management, the patient’s serum sodium levels gradually improved over the course of several days. Her inflammatory markers also showed progressive improvement, suggesting better control of the underlying infection, as shown in Table 1.

**Table 1:** Serial Laboratory Investigations Demonstrating Inflammatory Marker Improvement and the Correction of Hyponatremia During Hospital Admission. Serial laboratory investigations during the patient’s hospital admission demonstrating trends in inflammatory markers, electrolytes, and renal function. The table highlights the progressive reduction in C-reactive protein (CRP) levels, alongside the gradual correction of severe hyponatremia following the escalation of antibiotic therapy and the modification of fluid management. Renal function remained stable throughout the admission, with no significant deterioration in serum creatinine or estimated glomerular filtration rate (GFR). White cell count remained within the normal range, while mild anemia persisted during the course of hospitalization.

Test name	Measuring unit	Reference	11/03/2026	09/03/2026	06/03/2026	04/03/2026
White cell count	10^9^/L	3.7-11.0	8.8	8.6	9.1	10.1
Hemoglobin	g/L	115-165	107	105	111	95
Hematocrit	ratio	0.37-0.47	0.35	0.36	0.38	0.28
Mean cell volume	fL	76.0-100.0	84.3	84.8	87	86
Platelet count	10^9^/L	150-450	421	415	415	336
C-reactive protein	mg/L	0.0-10.0	42	125	196	301
Serum sodium	mmol/L	133-146	136	131	127	116
Serum potassium	mmol/L	3.5-5.3	4.4	4.2	4.2	4.6
Serum urea	mmol/L	2.5-7.8	4.5	4.5	4.8	4.7
Serum creatinine	μmol/L	44-97	41	43	46	46
Estimated GFR	mL/minute	-	>90	>90	84	86
Acute kidney injury	-	-	0	0	0	1

## Discussion

Hyponatremia in hospitalized patients is frequently multifactorial and can be particularly challenging to manage in the context of sepsis and pulmonary infection [5]. Pneumonia is a well-recognized trigger for hyponatremia, often through mechanisms involving inappropriate antidiuretic hormone secretion [6].

In this case, the patient presented with severe hyponatremia that persisted despite fluid restriction and evaluation for common endocrine causes. Investigations, including serum osmolality and cortisol levels, were normal, reducing the likelihood of adrenal insufficiency or other endocrine etiologies [7].

One possible contributing factor was ongoing systemic infection. Sepsis and inflammatory cytokines may stimulate the release of antidiuretic hormone, leading to impaired free water excretion and dilutional hyponatremia. The persistence of elevated C-reactive protein (CRP) levels suggested that the underlying infection had not been adequately controlled during initial antibiotic therapy [8].

The patient’s penicillin allergy limited antibiotic choices, resulting in the use of aztreonam. However, the inadequate response in inflammatory markers prompted escalation to meropenem. Following this change and the removal of fluid restriction, the patient’s sodium levels improved. This suggests that both persistent infection and fluid management strategies may have contributed to the electrolyte disturbance [9].

Fluid restriction is commonly employed in suspected syndrome of inappropriate antidiuretic hormone secretion (SIADH); however, inappropriate or prolonged restriction in certain clinical contexts may worsen patient outcomes, particularly when underlying sepsis or hypovolemia is present. Therefore, the careful reassessment of fluid management and underlying causes is essential in cases of refractory hyponatremia [10].

This case highlights the importance of considering ongoing infection as a driving factor for hyponatremia and emphasizes the need for a regular review of treatment strategies.

## Conclusions

Severe hyponatremia in hospitalized patients is often multifactorial and requires systematic evaluation. Pulmonary infections such as bronchopneumonia can contribute to hyponatremia through inflammatory and hormonal mechanisms. In this case, persistent infection and fluid restriction likely contributed to ongoing severe hyponatremia. The escalation of antibiotic therapy and the removal of fluid restriction were associated with the gradual normalization of sodium levels. Clinicians should maintain a high index of suspicion for unresolved infection and reassess fluid management strategies when treating refractory hyponatremia.
